# Effectiveness of psychotherapeutic consultation in the workplace: a controlled observational trial

**DOI:** 10.1186/s12889-016-3567-y

**Published:** 2016-08-26

**Authors:** Eva Rothermund, Harald Gündel, Edit Rottler, Michael Hölzer, Dorothea Mayer, Monika Rieger, Reinhold Kilian

**Affiliations:** 1 Department of Psychosomatic Medicine and Psychotherapy, University Hospital Ulm, Albert-Einstein-Allee 23, 89081 Ulm, Germany; 2 ZfP Suedwuerttemberg, Sonnenbergklinik, 70597 Stuttgart, Germany; 3 Health and Safety Sindelfingen, Daimler AG, 71059 Sindelfingen, Germany; 4 Institute for Occupational and Social Medicine and Health Services Research, University Clinic Tuebingen, Competence Centre Health Services Research, Medical Faculty Tuebingen, 72074 Tuebingen, Germany; 5 Department of Psychiatry II, University Hospital Ulm, BKH Guenzburg, 89312 Guenzburg, Germany

**Keywords:** Common mental disorder, Workplace based intervention, Psychotherapy, Mental health, Occupational psychiatry, Health services research

## Abstract

**Background:**

This study compares the effectiveness of psychotherapeutic consultation in the workplace (PSIW) with psychotherapeutic outpatient care (PSOC) in Germany.

**Methods:**

Work ability (WAI), quality of life (SF-12), clinical symptoms (PHQ) and work-related stress (MBI, IS) were assessed in 367 patients seeking mental health care via two routes (PSIW *n* = 174; PSOC *n* = 193) before consultation and 12 weeks later. Changes in outcome variables were assessed using covariance analysis with repeated measures (ANCOVA) with sociodemographic variables (propensity score method), therapy dose, setting and symptom severity as covariates.

**Results:**

The PSIW and PSOC groups included 122 and 66 men respectively. There were 102 first-time users of mental healthcare in the PSIW group and 83 in the PSOC group. There were group differences in outcome variables at baseline (*p* < 0.05); PSIW patients were less impaired overall.

There were no group difference in sociodemographic variables, number of sessions within the offer or symptom severity. There was no main effect of group on outcome variables and no group*time interaction. Work-related stress indicators did not change during the intervention, but work ability improved in both groups (*F* = 10.149, *p* = 0.002; baseline M = 27.2, SD = 8.85); follow-up M = 28.6, SD = 9.02), as did perceived mental health (SF-12 MCS), depression (PHQ-9) and anxiety (PHQ-7). Effect sizes were between η^2^ = 0.028 and η^2^ = 0.040.

**Conclusions:**

Psychotherapeutic consultation is similarly effective in improving patients’ functional and clinical status whether delivered in the workplace or in an outpatient clinic. Offering mental health services in the workplace makes it easier to reach patients at an earlier stage in their illness and thus enables provision of early and effective mental health care.

**Trial registration:**

DRKS00003184, retrospectively registered 13 January 2012.

## Background

Common mental disorders (CMD) will be one of the main contributors to the global economic burden of non-communicable diseases by 2030 [1]. Recently it has been anticipated that the direct and indirect costs of CMD amount to 3.5 % of the gross domestic product of industrial countries on average [2]. CMD and even sub-threshold mental health conditions have adverse effects on everyday life, work capacity and even mortality [3–6]. Because of their prevalence and costs CMD have an enormous impact on public health [7]. The increase in the prescription of psychotropic drugs, such as antidepressants, demonstrates that in practice this is the preferred approach to treatment, although it has been shown that a combination of psychotherapy and medication is the most effective treatment for most CMD [8, 9]. German guidelines recommend pharmacotherapy or psychotherapy as treatments for mild to moderate depressive disorders and explicitly recommend a combination of both for severe depression [10].

The treatment gap for CMD is estimated at 55 % globally while the improvement of treating CMD effectively has been considered to be very encouraging [11, 12]. Long waiting periods for initial consultations and psychotherapy are obstacles to appropriate treatment in healthcare systems across Europe, including in Belgium, Denmark, the Netherlands and Germany [13–17]. The interface between primary and secondary care may represent another barrier to effective treatment. In most countries general practitioners (GPs) mediate access to secondary services, i.e. specialist care. Unfortunately GPs often struggle to identify mental health problems [15, 18]. Fear of stigmatisation is another obstacle to timely treatment [19]. Thus there is a need to strengthen psychotherapeutic approaches by making them more accessible.

20 % of the working age population experience CMD at any one time [20]. The workplace has been identified as a key social context for early identification and treatment of mental health problems [2, 21, 22]. Focusing on what improves return to work after being sick listed due to depression [23] or on the effectiveness of work-directed interventions within the return to work process [24] restricted to randomised controlled trials showed positive but weak effects on work-related outcomes such as returning to work faster. Putting these findings together and including controlled trials Pomaki et al. [25] emerged important factors of how workplace-based interventions improve work disability outcomes: facilitation of access to clinical treatment, and availability of workplace-based psychotherapeutic interventions [25]. Chronic mental health problems, including subclinical symptoms, and impaired work ability are strong predictors of long sickness absence [26, 27]; this underlines the importance of early intervention.

Collaborative care offers (CCO) such as “psychotherapeutic consultation in the workplace” have been established as part of programmes to promote mental health in the workplace and are well-accepted by patients [28, 29].

Although the workplace environment is considered by many experts as an appropriate setting for a psychosocial intervention, there are currently no studies on the effectiveness of such interventions. A standard form of mental health treatment, short-term psychotherapeutic outpatient care (PSOC), has been adapted for delivery in the workplace under the label “psychotherapeutic consultation in the workplace” (PSIW) [30–32]. In this article we present the results of an observational controlled trial on the effectiveness of the PSIW program in comparison to the PSOC as a measure of routine care.

## Methods

### Study design

Due to reasons of the involved companies, staff policy and legal restrictions it was not possible to conduct a randomised controlled trial. Therefore, a prospective, controlled, observational trial comparing 174 employees from three companies who received PSIW with 193 outpatients from two clinics who received PSOC was conducted. Outcome indicators were assessed prior to the initial consultation (t1) and 12 weeks later (t2) [33].

### Study context

In the German healthcare system patients with CMD are treated by physicians specialising in psychiatry or psychosomatic medicine or by psychological psychotherapists. Treatment is usually delivered through private practices, the outpatient clinics of psychosomatic hospitals and psychosomatic departments or psychosomatic outpatient clinics at general hospitals [34]. In the German healthcare system PSOC is covered by statutory health insurance as well as by the private health insurance, and nearly 100 % of the population is covered by health insurance. Thus comprehensive care should be available to all those who need it [35]. In spite of this the treatment gap for CMD in Germany is comparable to that in other European countries [36–38].

### Intervention

#### Psychotherapeutic consultation in the workplace (PSIW)

PSIW is provided by a mental health expert (medical or psychological psychotherapist) who, although not employed by the host company, may be paid by the company (as an external contractor) or by the company’s health insurance funds. The service is available to all staff free of charge.

Staff members are usually informed about the service by the company physician. In one company employees must be referred to PSIW by the company physician but in others self-referral is possible. If self-referral is available information about PSIW (nature of the service; location; how to make an appointment) is communicated via paper and online.

In the first session the user’s clinical status and needs are being assed. This assessment is used to determine severity of the mental health problem and whether workplace consultation is a suitable treatment option or whether additional or more intensive mental health care (e.g. outpatient psychotherapy; short-term (8–10 sessions) psychotherapy; psychopharmacological treatment; inpatient treatment) is needed. Each session lasts 50–60 minutes and a maximum of four sessions can be offered under the PSIW programme.

After assessment the PSIW user will be informed about any further therapeutic steps that are indicated, motivating the service user to obtain additional help and providing specific advice to help individuals overcome barriers to service use. This involves providing the PSIW user with information about CMD and psychotherapeutic approaches to treatment. The strengths and resources of the patient are stressed and further treatments are recommended. If appropriate information about self-help books, counselling centres, e.g. family or drug and addiction counselling, and other services such as workshops on relaxation techniques is provided. Recommendations are made according to the clinical guidelines of the Association of the Scientific Medical Societies in Germany (AWMF) as they exist e.g. for depression [10].

PSIW takes place only in companies that agree to offer the service and provide structural support. Company support includes appointment making and providing a suitable location for consultations. Subject to patient consent the next steps in treatment are determined in a case conference involving the occupational health physicians and the psychotherapeutic consultant.

#### Psychotherapeutic outpatient care (PSOC)

Initial PSOC treatment is limited to two sessions and the core elements are assessment of clinical symptoms and service needs, provision of information about CMD and treatment methods and recommendations for further treatment. Referral to PSOC is predominantly via GPs. Self-referral is encouraged and information for patients drafted via the clinic websites.

### Eligibility criteria and recruitment process

To be eligible for the study participants had to be at least 18 years old, capable of understanding and writing German and currently employed. Participants in the PSIW group had to be employed by one of the participating companies.

Participants in the PSOC group were recruited consecutively from two outpatient clinics, the University Clinic of Psychosomatic Medicine and Psychotherapy, Ulm, and Sonnenbergklinik, Division of Psychosomatic Medicine of the ZfP Suedwuerttemberg, Stuttgart, from June 2012 to January 2013.

Participants in the PSIW group were recruited consecutively from November 2011 to June 2013.

### Assessment

Assessment of clinical status was made by means of self-administrated questionnaires (Table 1). Questionnaires for baseline assessment were handed over at the first meeting. Follow-up questionnaires were sent out by mail. Data were double entered manually and checked twice.

#### Work ability

Ability to work was the primary outcome indicator and was assessed using the work ability index (WAI), a self-report instrument used to assess current and future work ability and work demand management based on behavioural measures [39].

#### Use of mental health services and symptom duration

Lifetime history of psychotherapeutic treatment and number of contacts with mental health services over the previous 12 months were recorded. Duration (in months) of the symptoms which had prompted the consultation was recorded by the therapist.

#### Mental health and somatic symptoms

Depression was assessed with the 9-item Patient Health Questionnaire - depression scale (PHQ-9) [40], anxiety with the 7-item Patient Health Questionnaire - generalised anxiety disorder scale (PHQ-7) [41] and somatic symptom severity with the 15-item Patient Health Questionnaire - somatisation (PHQ-15) [42]. Interpretation of PHQ scores was based on the diagnostic criteria of the DSM-IV and ICD-10, using the recommended cut-off of 10 or above to distinguish between clinical and non-clinical levels of symptoms [43].

#### Health-related quality of life

Health-related quality of life was assessed using the SF-12, a validated, short version of the SF-36, to measure the functional health status of patients [44]. Weighted summation provided summary scores for perceived mental health (MCS; mental health component score) and perceived physical health (PCS; physical health component score).

#### Work-related stress

We assessed work-related stress using the German version (MBI-GS-D) of the Maslach Burnout Inventory (MBI), which is used to assess burnout syndrome as a manifestation of mental exhaustion [45]. The three components of burnout are: emotional exhaustion (EE), depersonalisation (DP) and reduced personal accomplishment (PA). Respondents indicate the frequency with which they experience each item. Sum scores were used instead of means for each subscale.

#### Irritation

Irritation is defined as subjectively perceived emotional and cognitive strain in occupational contexts [46, 47]. We assessed irritation with the irritation scale (IS).

### Statistical analyses

#### Sample size determination

We calculated that a sample of 220 participants would be needed to detect a medium (effect size *f* = 0.25) difference in WAI with a power of 0.95 at a significance level of *p* < 0.05 using repeated measures ANOVA.

#### Bias control

The propensity adjustment method was used for bias control [48]. Propensity scores were estimated as the conditional probability of belonging to the PSIW group using a logistic regression model including age, gender, family status (marital status, living with children or not, living with other adults or not), job status (school-leaving qualification, full or part-time employment, occupation: uneducated, skilled or white collar worker, managerial staff), duration of psychopathological symptoms, history of psychiatric or psychotherapeutic treatment (outpatient treatment: lifetime, previous 12-month, inpatient treatment: lifetime, previous 12-month, other physician’s care: previous 12-month, psychosocial treatment within company: previous 12-month, other like pastoral advice service etc.), and baseline clinical differences as independent variables. Number of sessions and presence of treatment between the first consultation and follow-up assessment were included in the model as covariates.

Latent profile analysis (LPA) [49] including the WAI, PHQ-9 and the SF-12 was used to provide an overall indication of clinical differences at baseline. Details of the LPA procedure and results are published elsewhere (Rothermund et al. submitted). On the basis of the LPA participants were classified into four classes (1 = severely mentally ill, 2 = moderately affected with low quality of life, 3 = moderately affected with low work ability, 4 = less affected but at risk) which captured global differences in baseline clinical measures. Class membership was included in the logistic regression model as a categorical independent variable with class four as the reference category.

#### Effectiveness analysis

The effectiveness of the intervention was estimated with respect to primary and secondary outcomes on an intention to treat (ITT) basis. Missing endpoint data were imputed as last observation carried forward (LOCF).

Change in outcome indicators was analysed by covariance analysis with repeated measures (ANCOVA) with propensity scores as covariates. Statistical analyses were performed using SPSS version 21.

## Results

**Fig. 1:**
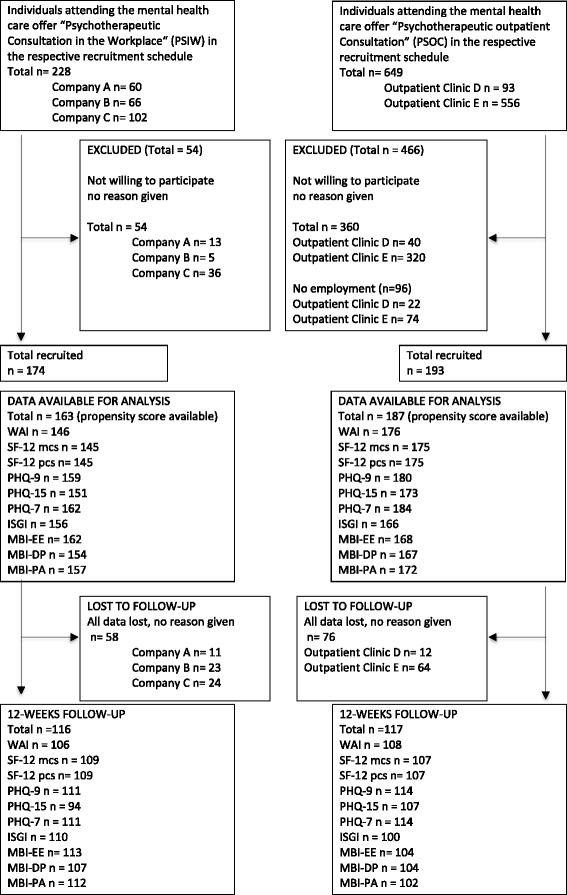
Flow diagram showing participants numbers at different stages

A total of 367 participants were included in the analyses (PSIW *n* = 174; PSOC *n* = 193) (see flow diagram, Fig. 1 and Table 2 for sociodemographic data). Due to missing propensity scores a total of 17 cases (4.6 %) was excluded from the analyses. Follow-up data were obtained of 60 % of participants. Reasons for dropout were not given. Non-respondents were younger (38, SD 11.3) than respondents (45, SD 10.7). No significant differences were found for gender, educational level, symptom severity or any of the psychometric measures. The PSIW group was slightly older (45 years, SD = 10.1) than the comparison group (40 years, SD = 12.1) and PSIW participants tended to have been experiencing symptoms for a shorter period of time (38 months, SD = 65.4) than PSOC participants (51 months, SD = 72.9). The majority of the PSIW group were men (*n* = 122, 70 %) and just over a third of the PSOC group was men (*n* = 66, 34 %). The PSIW group contained 102 first-time users of mental health services (65 %); the corresponding figure for the PSOC group was 83 (45 %). 12-month usage of mental health services (number of participants who had at least one contact with mental health services) was lower in the PSIW group (*n* = 65) than the PSOC group (*n* = 119).

**Table 1:** Measures and instruments used

Measure	Instrument	Range	Category boundaries/interpretation				Source
WAI	work ability index	7–49	49–44 very good work ability	43–37 good work ability	36–28 moderate work ability	27–7 very low work ability	[64]
SF-12-MCS	SF-12-mental component score	0–100	to compare: normative German sample 1994: 51.2, psychosomatic inpatients 27				[44, 65]
SF-12-pcs	SF-12-physical component score	0–100	to compare: normative German sample 1994: 46.3, psychosomatic inpatients 40				
PHQ-9-depr	patient health questionnaire depression	0–27	0 to 4 minimal symptom burden	5 to 9 mild symptom burden	10 to 14 moderate symptom burden	>15 severe symptom burden	[43]
PHQ-15-som	patient health questionnaire somatoform symptom severity	0–30					
PHQ-7-anx	patient health questionnaire anxiety	0–21					
ISGI	irritation scale global index	8–56	no irritation 8–16	low irritation 17–26	moderate irritation 27–38	strong irritation 39–56	[46]
MBI-EE	burnout-emotional exhaustion	5–30	<17.5 unremarkable	>17.5 alerting	>25 critical		[45]
MBI-DP	burnout- depersonalisation	5–30	low scores are healthier				
MBI-PA	burnout- personal accomplishment	6–36	high scores are healthier

**Table 2:** Sample description

Characteristic		total sample	PSIW	PSOC	*P* value
		*n* = 367	*n* = 174	*n* = 193	
age (years)	mean (SD)	42.94 (11.47)	45.20 (10.12)	40.05 (12.07)	*** b
symptom duration (months)	mean (SD)	44.94 (69.68)	38.02 (65.41)	51.06 (72.87)	n.s. b
male gender	n (%)	188 (51.2)	122 (70.1)	66 (34.2)	*** a
in a stable relationship	n (%)	239 (68.5)	126 (75.9)	113 (61.7)	* a
school-leaving qualification	n (%)	364 (100)	171 (100)	193 (100)	
still at school		4 (1.1)	1 (0.6)	3 (1.6)	n.s. a
low (9 years)		106 (29.1)	57 (33.3)	49 (25.4)	
medium (10 years)		133 (36.6)	55 (32.2)	78 (40.4)	
high (<10 years)		121 (33.2)	58 (33.9)	63 (32.6)	
first-time user (no previous in- or out-patient treatment)	n (%)	185 (54.1)	102 (65.4)	83 (44.6)	***a
mental health care system user in the previous 12 months	n (%)	184 (50.1)	65 (37.4)	119 (61.7)	***a

*SD* standard deviation, *n* number, *n.s.* not significant, *a* chi-square test, *b t*-test

* *p* < 0.05, ** *p* < 0.01, *** *p* < 0.001

**Table 3:** Effects of the PSIW and PSOC interventions on work ability and clinical and work-related parameters at baseline and follow-up

Variable	Group	Baseline mean (SD)	Follow-up mean (SD)	Group effect, *F*-value, p, η^2^	Time effect *F*-value, p, η^2^	Group*Time effect, *F*-value, p, η^2^
WAI	PSIW	29.5 (8.02)	30.8 (8.32)	(1:317) = 1.832, 0.177, 0.006	(1:317) = 10.149, 0.002, 0.031	(1:317) = 1.602, 0.206, 0.005
	PSOC	25.3 (9.07)	26.8 (9.21)			
	all	27.2 (8.85)	28.6 (9.02)			
	^a^Δ PSIW vs. PSOC	*p* = 0.000				
SF-12 MCS	PSIW	33.3 (11.13)	37.5 (11.77)	(1:315) = 0.155, 0.694, 0.000	(1:315) = 9.093, 0.003, 0.028	(1:315) = 6.950, 0.009, 0.022
	PSOC	29.8 (10.24)	32.7 (11.07)			
	all	31.4 (10.78)	34.9 (11.62)			
	^a^Δ PSIW vs. PSOC	*p* = 0.002				
SF-12 PCS	PSIW	45.8 (11.13)	45.4 (10.96)	(1:315) = 0.321, 0.572, 0.001	(1:315) = 0.858, 0.355, 0.003	(1:315) = 0.679, 0.411, 0.002
	PSOC	43.8 (11.31)	44.5 (10.90)			
	all	44.7 (11.36)	45.0 (10.92)			
	^a^Δ PSIW vs. PSOC	*p* = 0.146				
PHQ-9	PSIW	11.2 (5.71)	9.6 (6.05)	(1:334) = 0.600, 0.439, 0.002	(1:334) = 13.946, 0.000, 0.040	(1:334) = 1.821, 0.178, 0.005
	PSOC	14.4 (6.24)	12.4 (6.44)			
	all	12.9 (6.20)	11.1 (6.40)			
	^a^Δ PSIW vs. PSOC	*p* = 0.000				
PHQ-15	PSIW	10.5 (5.41)	9.7 (5.44)	(1:319) = 0.527, 0.468, 0.002	(1:319) = 3.444, 0.064, 0.011	(1:319) = 1.891, 0.170, 0.006
	PSOC	11.4 (5.44)	10.9 (5.56)			
	all	11.0 (5.44)	10.3 (5.53)			
	^a^Δ PSIW vs. PSOC	*p* = 0.196				
PHQ-7	PSIW	9.5 (5.36)	8.2 (5.31)	(1:341) = 0.500, 0.480, 0.001	(1:341) = 12.259, 0.001, 0.035	(1:341) = 0.858, 0.355, 0.003
	PSOC	11.3 (5.53)	9.5 (5.43)			
	all	10.4 (5.50)	8.9 (5.41)			
	^a^Δ PSIW vs. PSOC	*p* = 0.005				
IS	PSIW	30.7 (11.74)	29.7 (12.23)	(1:317) = 1.453, 0.229, 0.005	(1:317) = 2.243, 0.135, 0.007	(1:317) = 1.719, 0.147, 0.007
	PSOC	35.2 (12.12)	34.2 (12.33)			
	all	33.0 (12.13)	32.1 (12.47)			
	^a^Δ PSIW vs. PSOC	*p* = 0.001				
MBI-EE	PSIW	3.9 (1.27)	3.8 (1.27)	(1:325) = 0.012, 0.912, 0.000	(1:325) = 4.199, 0.041, 0.013	(1:325) = 0.758, 0.385, 0.002
	PSOC	4.5 (1.24)	4.3 (1.18)			
	all	4.2 (1.28)	4.1 (1.25)			
	^a^Δ PSIW vs. PSOC	*p* = 0.000				
MBI-DP	PSIW	3.2 (1.11)	3.2 (1.10)	(1:316) = 0.150, 0.699, 0.000	(1:316) = 0.524, 0.470, 0.002	(1:316) = 0.008, 0.927, 0.000
	PSOC	3.5 (1.22)	3.5 (1.18)			
	all	3.3 (1.17)	3.4 (1.15)			
	^a^Δ PSIW vs. PSOC	*p* = 0.015				
MBI-PA	PSIW	4.5 (0.82)	4.5 (0.78)	(1:324) = 0.760, 0.384, 0.002	(1:324) = 0.437, 0.509, 0.001	(1:324) = 0.001, 0.980, 0.000
	PSOC	4.4 (0.91)	4.3 (0.92)			
	all	4.4 (0.86)	4.4 (0.86)			
	^a^Δ PSIW vs. PSOC	*p* = 0.115				

*Legend*: ^a^ Δ PSIW vs. PSOC (p): Results of a *t*-test comparing PSIW and PSOC groups at baseline, *η* ^2^ partial square eta (effect size), *p* level of significance

At two companies most referrals to PSIW were made by occupational physicians or social workers; at the third company employees were usually self-referred. For a more detailed description of procedures of PSIW see Rothermund et al. 2014 [31].

### Baseline data (Table 3)

At baseline the PSIW group had higher work ability (M = 29.5, SD = 8.02) than the PSOC group (M = 25.3, SD = 9.07; *p* < 0.001). The PSIW group also had better perceived mental health (SF-12 MCS: M = 33.3, SD = 11.13) than the PSOC group (M = 29.8, SD = 10.24, *p* = 0.002) at baseline. Depression scores at baseline were also lower in the PSIW group (M = 11.3, SD = 5.72) than the PSOC group (M = 14.5, SD = 6.28, *p* < 0.001). Anxiety scores showed a similar pattern (PSIW group: M = 9.6, SD = 5.36; PSOC group: M = 11.2, SD = 5.71; *p* < 0.001). There were no group differences in physical health (SF-12 PCS) and somatic symptom severity (PHQ-15) at baseline.

Work-related stress was also lower in the PSIW group at baseline. IS scores were lower in the PSIW group (M = 30.7, SD = 11.74) than the PSOC group (M = 35.2, SD = 12.12, *p* = 0.001). The PSIW group also had lower scores on two aspects of burnout. EE was lower in the PSIW group (M = 3.9, SD = 1.27) than in the PSOC group (M = 4.5, SD = 1.24; *p* < 0.001) and the PSIW group showed less DP (M = 3.2, SD = 1.11) than the PSOC group (M = 3.5, SD = 1.22; *p* = 0.015). There was no baseline group difference in PA.

Overall, patients that were recruited in the workplace context (PSIW group) appeared to be healthier than patients that were using established services.

### Effects of PSIW and PSOC on work ability and clinical and work-related parameters (Table 3)

Both groups showed a similar improvement in work ability over time (baseline M = 27.2, SD = 8.85; follow-up M = 28.6, SD = 9.02; *F* = 10.149, *p* = 0.002). At follow-up the mean work ability of the PSIW group (M = 30.8, SD = 8.32) was higher than that of the PSOC group (M = 26.8, SD = 9.21). However there was no group effect and no group*time interaction for WAI score. None of the 3 covariates (propensity score; number of sessions; presence of further treatment) had an effect on WAI.

Both groups showed similar improvements in perceived mental health (SF-12 MCS; baseline M = 31.4, SD = 10.78; follow-up M = 34.9, SD = 11.62; *F* = 9.093, *p* = 0.003), depression (PHQ-9; baseline M = 12.9, SD = 6.20; follow-up M = 11.1, SD = 6.40; *F* = 13.946, *p* < 0.001) and anxiety (PHQ-7; baseline M = 10.4, SD = 5.50; follow-up M = 8.9, SD = 5.41; *F* = 12.259, *p* = 0.001). The effect sizes for the changes were small (η^**2**^ between 0.028 and 0.040). There was no effect of group and no group*time interaction on these outcome parameters.

There were no changes in measures of physical health (perceived physical health measured as SF-12 PCS score; somatoform symptoms, measured as PHQ-15 score) or work-related stress (all aspects of burnout: EE, DP and PA; IS score).

## Discussion

In this trial we assessed work ability, quality of life, clinical symptoms and work-related stress in 367 patients who received mental health services via two programmes, PSIW and PSOC. Patients were assessed before their first consultation and 12 weeks later and in the analyses we controlled for variance in sociodemographic factors, therapy dose, setting and symptom severity.

Our results indicate that psychotherapeutic consultation is similarly effective in improving patients’ functional and clinical status whether delivered in the workplace or in a psychotherapeutic outpatient clinic. Our data clearly suggest that workplace-based services reach people with mental health problems at an earlier state of illness than the traditional health care system. Thus, psychotherapeutic care in an occupational healthcare setting can be considered an early and effective form of mental health care.

The work ability of individuals improved after the initial intervention independent of the setting in which patients sought help; this was a small effect (baseline WAI: M = 27.2 points, SD = 8.02; follow-up WAI: M = 28.6, SD = 9.02; η^2^ = 0.031; *p* = 0.002). The same pattern of improvement was observed for depression (baseline PHQ-9: M = 12.9, SD = 6.20; follow-up PHQ-9: M = 11.1, SD = 6.40; *F* = 13.946, *p* < 0.001) and anxiety (baseline PHQ-7: M = 10.4, SD = 5.50; follow-up PHQ-7: M = 8.9, SD = 5.41; *F* = 12.259, *p* = 0.001). Perceived mental health (SF-12 MCS), also known as health related quality of life, also improved (baseline M = 31.4, SD = 10.78; follow-up M = 34.9, SD = 11.62; *F* = 9.093, *p* = 0.003). Effect sizes for all improvements were small, between η^2^ = 0.028 and η^2^ = 0.040.

The small effects are plausible as the interventions were very short: one or two sessions, primarily focusing on diagnostic assessment and facilitation of access to other clinical services. A recent study showed that better work ability at baseline was associated with better outcomes, e.g. early return to work [27]. This study focused on short time effects, as the follow-up assessment occurred 12 weeks after the initial consultation. This limitation may have obscured better outcomes in the group treated in the workplace due to the earlier detection of illness. Nevertheless the parallel improvement of clinical parameters and work ability is meaningful, as we know that recovery from depression does not automatically mean that a person is once more capable of dealing with the demands of everyday life [50–52].

One might have expected the PSIW group to show less spontaneous improvement than the PSOC group on the grounds that they were healthier and would therefore show a smaller response to the initial very short, primarily diagnostic intervention offered. Alternatively one might expect the PSIW group to show greater improvement as prognosis for mental health conditions is usually better in cases where intervention is provided early in the course of disease [53–55].

The findings of this study are in line with findings on return to work process following CMD-related absence. Vlasveld et al. 2012 [56] investigated employees absent from work with major depressive disorder and found that collaborative care produced similar effects to care as usual when outcome was measured as a continuous variable with PHQ-9; however the response rate was higher in the collaborative care group. This probably reflects an aspect of the collaborative care which was not captured in the analyses. Van der Feltz-Cornelis et al. 2010 [57] showed that return to work was faster in an intervention group than a group who received care as usual. The intervention consisted of treatment by occupational physicians who were advised by psychiatric consultants. Both groups showed comparable improvements in symptom severity and quality of life.

During the 12-week period between first consultation and follow-up assessment there was no change in work-related stress (irritation, burnout) in either group, although there were improvements in overall mental well-being, depression and anxiety. There were also no changes on the scales measuring aspects of physical health, namely somatoform symptoms (PHQ-15) and perceived physical health (SF-12 PCS). The lack of effects on work-related stress and physical health may have been because the PSIW programme did not target work-related health or physical health but focused on diagnostic assessment and motivation for further psychotherapeutic treatment.

### Limitations and strengths

Our observation that the group accessing psychotherapeutic services in the workplace did not improve more than those accessing services via conventional outpatient clinics may have been influenced by the short length of the observation period of only 12 weeks. We know from research on maintaining gains from psychotherapy that psychotherapeutic interventions may have long term effects and hence follow-up for at least at 12 month after intervention is recommended [58–60]. Our study also suffers from the familiar limitations associated with lack of a randomised group assignment. Thus, bias control was addressed using the propensity score method [61] with only rare (max. 6 %) reduction of sample size. The controlled observational design allowed us to manage the problem of artificial deterioration of the sample. Randomised controlled trials may suffer deterioration of the sample, due to the better control of intervening variables, which often leads to homogenous samples that differ from samples under routine conditions [60, 62, 63]. In our study the intervention was studied under routine conditions and thus the study has good external validity and the findings should be generalizable.

A loss to follow-up of 40 % is quite high. The only detectable difference between non-respondents and respondents was age (38, SD 11.3 vs 45, SD 10.7 years). Thus we conclude the results might not be generalizable to younger workers.

Another limitation is that we did not collect data on therapeutic recommendations and whether they were followed. Such data would have helped us to interpret the pattern of changes between the baseline and follow-up assessment and will therefore be collected and analysed in our next investigation. Finally, the sample was drawn from a single region of Germany rather than from across Europe and so the findings should only be generalised to other countries with different healthcare systems with caution.

## Conclusions

We conclude that the PSIW intervention is effective over a 3 month-follow-up period, covering diagnostic assessment and the very early psychotherapeutic sessions. The effects of the intervention were most marked with respect to social functioning (work ability) and mental health in patients with sub-syndromal illness and CMD. To make the intervention more effective in this setting than it is in its current form it would need to be tailored even more to the specific needs of the target group. Exhaustion is one of the major topics mentioned as reason for utilising PSIW (qualitative data in preparation for publication). Ongoing interpersonal conflicts, problems regulating demands and efforts, or problems in prioritising are likely drivers for exhaustion on the individual side. On the organisational side working conditions may contribute to this problem. A short time psychotherapeutic intervention (up to 10 sessions) should be offered to detect relevant areas of conflict and to consequently analyse and address specific individual and/or organisational reasons in more detail. Together with consecutive motivation for further clinical treatment if necessary this approach should make a short-time intervention even more powerful.

Showing that the intervention is effective in the new setting workplace while reaching different users especially due to gender and course of disease drafts the need for in depth information about: which ingredients, barriers, drivers and synergies can be identified in that kind of health care offer in the workplace? We are currently analysing qualitative data which address these questions.

## Abbreviations

ANCOVA: Covariance analysis with repeated measures
AWMF: Association of the scientific medical societies in Germany
CCO: Collaborative care offers
CMD: Common mental disorder
DP: Depersonalisation
EE: Emotional exhaustion
GP: General practitioner
IS: Irritation scale
ITT: Intention to treat
LOCF: Last observation carried forward
LPA: Latent profile analysis
M: Mean
MBI: Maslach burnout inventory
N: Number
n.s.: Not significant
PA: Depersonalisation
PHQ-7/-9/-15: Patient health questionnaire 7 items generalised anxiety/9 items depression/15 items somatisation
PSIW: Psychotherapeutic consultation in the workplace
PSOC: Psychotherapeutic outpatient care
SD: Standard deviation
SF-12 mcs/pcs: Short form-12 Items mental health component score/physical health component score
WA: Work ability
WAI: Work ability index
